# Does surgery and radiotherapy really improve the prognosis of patients with small-cell esophageal carcinoma?

**DOI:** 10.1097/JS9.0000000000001020

**Published:** 2023-12-19

**Authors:** Kexun Li, Jie Mao, Yunchao Huang

**Affiliations:** Department of Thoracic Surgery I, Key Laboratory of Lung Cancer of Yunnan Province, Yunnan Cancer Hospital, The Third Affiliated Hospital of Kunming Medical University, Cancer Center of Yunnan Province, Kunming, People’s Republic of China

*Dear Editor,*

We have read with great interest a paper titled ‘Surgery versus radiotherapy for limited-stage small cell esophageal carcinoma: a multicenter, retrospective, cohort study in China’ published in the *International Journal of Surgery* by Zhu *et al*.^1^. In this study, the authors conducted a large, multicenter cohort study and utilized univariate and multivariate analysis along with propensity score matching (PSM) to adjust for confounding factors. They compared the overall survival (OS) and progression-free survival (PFS) differences between groups using K–M curves. The results showed that both surgery and radiotherapy (RT) significantly improved the OS and PFS of patients with limited-stage small cell esophageal carcinoma (LS-SCEC) when compared to only chemotherapy (CT). Although this study has several strengths, there are certain points that require further discussion.

Firstly, the study included 142 patients with stages I and II, 17 (12.0%) under CT alone, 36 (25.4%) under CT combined with RT, yet surgical treatment should be considered as optimal treatment strategies for AJCC (American Joint Committee on Cancer) staging stages I and IIA^2,3^, which may limit the study and affect the conclusions.

Secondly, in the method section (Statistical analysis), you defined the statistical significance as a *P* value <0.05 in a two-tailed test. However, even after performing PSM, there was still a statistically significant difference (*P*=0.04) in the CT regimen between the CT+RT and CT+S groups. Furthermore, in the univariate and multivariate Cox analysis for CT+S vs. CT+RT, the CT regimen was identified as an important factor affecting the PFS hazard ratio (HR) and OS HR. Therefore, it is valuable to further explore and analyze the confounding factors between them for optimizing the prognostic model and improving the survival prognosis of patients.

In the univariate and multivariate analysis of this study, several factors were found to have statistical significance for Cox of OS HR and PFS HR, including Karnofsky performance status (KPS), TNM stage, Treatment, and CT cycle. Additionally, there were numerous statistically different factors among the three groups in terms of clinicopathological characteristics of LS-SCEC, such as age, TNM stage, and CT cycle. Previous studies have also shown that age and TNM stage can impact the survival of patients with esophageal cancer^4,5^. Notably, the proportion of patients older than 70 in the CT alone group (31%) exceeded that of the other two groups, as did the TNM stage factor in 4a (20/23.8%). The results section compared the OS and PFS, revealing that patients in the CT+RT and CT+S groups had significantly better outcomes (*P*<0.01) in terms of OS and PFS compared to the CT alone group. It is important to acknowledge that there are still significant differences in the aforementioned factors affecting survival, and conducting PSM could further enhance the credibility of the results.

## Ethical approval

None.

## Sources of funding

The study was funded by National Natural Science Foundation of China (81960335); National Clinical Key Specialty Construction Project (Thoracic surgery construction project); Science and Technology Department of Yunnan Province (Yunnan Province of Li Yin specialist workstation).

## Author contribution

K.L.: drafting of the article and statistical analysis; Y.H. and J.M.: administrative, technical, or material support and obtained funding; K.L., J.M., and Y.H.: had full access to all the data in the study and take responsibility for the integrity of the data and the accuracy of the data analysis. All authors were involved in the study concept and design, acquisition, analysis, or interpretation of data.

## Conflicts of interest disclosure

The authors have no conflicts of interest to declare.

## Research registration unique identifying number (UIN)

None.

## Guarantor

Kexun Li and Yunchao Huang are guarantors.

## Unblinded ethics statement

None.
